# How to Achieve Universal Health Coverage: A Case Study of Uganda Using the Political Process Model: Comment on "Health Coverage and Financial Protection in Uganda: A Political Economy Perspective"

**DOI:** 10.34172/ijhpm.2022.7307

**Published:** 2022-06-21

**Authors:** Cidalia Eusebio, Maria Bakola, David Stuckler

**Affiliations:** ^1^Independent Researcher, London, UK.; ^2^Research Unit for General Medicine and Primary Health Care, Faculty of Medicine, School of Health Science, University of Ioannina, Ioannina, Greece.; ^3^Dondena Centre for Research on Social and Population Dynamics, Milan, Italy.; ^4^Cergas Centre for Research on Health and Social Care Management, Milan, Italy.; ^5^Department of Social & Political Sciences, Bocconi University, Milan, Italy.

**Keywords:** Universal Health Coverage, Low Income Countries, Middle Income Countries, Political Economy, Uganda

## Abstract

How can resource-deprived countries accelerate progress towards universal health coverage (UHC)? Here we extend the analysis of Nanini and colleagues to investigate a case-study of Uganda, where despite high-level commitments, health system priority and funding has shrunk over the past two decades. We draw on the Stuckler-McKee adapted Political Process model to evaluate three forces for effecting change: reframing the debate; acting on political windows of opportunity; and mobilising resources. Our analysis proposes a series of pragmatic steps from academics, non-governmental organisations, and government officials that can help neutralise the forces that oppose UHC and overcome fragmentation of the pro-UHC movement.

## Introduction

 In their recent analysis, “Health Coverage and Financial Protection in Uganda: A Political Economy Perspective” Nanini and colleagues^1^ aim to understand the political forces at play in Uganda’s quest to achieve universal health coverage (UHC). Based on interviews and an analysis of governmental documentation, they conclude that the current political climate is unfavourable, for two reasons: the lack of a national strategy and the presence of multiple competing interests.

 Yet it is clear that something needs to change. In Uganda, health spending dropped from 5.1% of gross domestic product in 2000 to 3.8% in 2019. This reveals a falling priority placed on health by the government and a failure to reach the globally recognised target of at least 5%.^2^ Although Uganda officially abolished user fees for healthcare in 2001, high out-of-pocket payments persist, reflecting the role of informal payments. In addition, the perceived lower quality of public healthcare in Uganda lead many people to seek care from the private sector despite higher out-of-pocket costs.^3^

 Perhaps counterintuitively, this reversal of progress towards UHC is happening despite Uganda’s high-level commitment towards the Sustainable Development Goals (SDGs), including those placing priority on UHC. By UHC, we refer to attaining “access to the full range of essential health services, such as health promotion, prevention, treatment, rehabilitation and palliative care, from all people, when and where they need them, without any financial difficulty.”^4^ Achieving financial protection is critical for Uganda, and systematic reviews have demonstrated that lowering out of pocket costs can substantially improve health.^5^

 So what can be done in this context where UHC, while ostensibly an agreed upon goal of the government and UN, seems to be slipping further from reach?

 We applaud Nanini and colleagues’ emphasis on the political economy and external health system factors. We also second their recommendation to enshrine a commitment to UHC in legislation. Yet to realise this commitment, we must go beyond their analysis and delve deeper into analysing the pro-UHC forces. To do so here we draw on the Stuckler-McKee adapted Political Process model to understand how to effect real change in Uganda’s context. This model has been previously used to evaluate social movements in global health and chronic noncommunicable diseases, among others.^6^ It involves three processes that can converge to achieve to real change: (*i*) reframing the debate on UHC, (*ii*) identifying and creating political opportunities, and (*iii*) mobilizing resources for change.^7^ We cover each in turn.

## Reframing the Debate: UHC as a Unifying and Equalising Force

 Reframing the debate is often a critical step to effecting change. It starts by changing the way a political issue is discussed. Those who control the ‘frame of the debate’ often control what is not only on the agenda politically, but what is off. Often politicians might be faced with opponents’ frames and in this case, they will try to reframe the debate in order to better communicate their message and convince their audience.^8^

 At least two important frames have been used in the quest to achieve UHC historically in developing countries and could be leveraged in Uganda’s current efforts to attain it. One is to frame UHC as ‘nation-building’ – helping unify and heal fractures that exist across ethnicities, social classes and other socio-political cleavages to create a unified national identity.^7^ Historically countries as diverse as Germany and South Korea used UHC as a unifying rhetoric; more recently countries like Rwanda helped accelerate their progress to UHC in similar ways. Part of the framing adopted, “Mutuelle de Santé,” was not only linked to unifying a fractured society, but also to advancing development to become a middle-income country.^9^ Here, we argue that Uganda’s political ambition to become a middle-income country creates a powerful opportunity to make the case for how attaining UHC could boost economic growth. This framing could draw on the now established body of evidence on how investing in health offers one of the most attractive fiscal multipliers, achieving as much as $3 return on every $1 invested.^10^

 Another is to shine a spotlight on how Uganda’s non-universal system is failing vulnerable groups, particularly children, elderly and pregnant women. These groups have high levels of ‘desert’ in politics and as such tend to make for powerful framing, as has commonly been deployed on issues that have strong opposition, such as tobacco and unhealthy foods. Here academic partners, ideally in partnership with the health ministry and/or non-governmental organisations, can play a key role to quantify the avoidable health inequalities that arise from the lack of financial protection in Uganda. This could be particularly powerful in associated with coronavirus disease 2019 (COVID-19), showing how the pandemic has caused a double-burden of disease and impoverishment among those who do not benefit from UHC.

## Identifying and Creating Political Opportunities: The 2030 Agenda and the COVID-19 Pandemic

 Big political changes happen infrequently. Nannini and colleagues note that maintaining status quo will not be enough to achieve UHC. For a system-wide transformation, often there is a need to go beyond politics as usual. These situations can be triggered by major events or structural changes. These include events like natural disasters, such as earthquakes and cyclones, or structural changes, like economic crises or government transitions. They can also include international policies and programmes which open up time-limited ‘political windows of opportunity’ to effect real change.

 Two such opportunities are present now in Uganda. First, the United Nations’ 2030 agenda, a movement to accelerate progress towards SDG. This differs from the SGD itself, as it offers specific target milestones, which can help setting actionable goals towards UHC.^11^

 Second, having successfully encountered several outbreaks in the last few years and the experiences gained from them, helped Uganda to create a COVID-19 preparedness and response plan. This is an opportunity to improve multi-sectoral collaboration within the government, strengthen the collaboration with the private sector and civil society, enhance the community involvement and participation and universalize the entire health system.^12^

## Mobilizing the Resources: Balancing the Entrenched Power of the Private Sector

 Inevitably slow progress in Uganda to UHC, against a backdrop of high-level commitments and supports, reflects a reality that there are those who stand to lose. The important paper by Nannini and colleagues observes that the government appears to have shifted national priorities to the sectors perceived to be productive engines of the economy and, in so doing, the government seems to have little or no interest in being the first player in the provision of health services and financing. However, they have yet to address the important role of vested interests who may have been behind this abdication of responsibility, including, among others, the powerful private sector in health.

 Without mobilising resources to back a pro-UHC movement and hold government accountable it is impossible to capitalise on political windows of opportunity. Most recently, for example, the National Resistance Movement party won the democratic election in 2021, campaigning on a platform that included introducing a legislative commitment to UHC with the slogan “free healthcare.”^1^ Yet once attaining power, the ideology changed towards the supremacy of the market forces, without being accountable electorally.^1^

 At present there is a stark imbalance between the concentrated political power of the private sector in healthcare, versus the fragmented and diffuse social-forces of communities, advocacy groups, and people who would benefit from UHC. Government officials have, perhaps perversely, tended to rely on external donors for both funding and leadership, without the necessary accountability and commitment for UHC. This also can create an adverse situation where development partners come to believe that health services have been delegated to them.

 In Uganda there are major gaps in the movement for UHC. Communities are proactively collaborating to build UHC, by enabling community health insurance systems, but they are fragmented, without a national strategy and leadership. Despite the inefficiencies and inequalities of such health insurance, we believe it is creating a political opportunity. Not only local ownership is important for successful implementation,^13^ but also to achieve SDG.

 Current initiatives are underway to improve accountability, collaboration and community ownership although their sustainability is uncertain. One of those examples is the African Collaborative for Health Financing Solutions. The initiative has formed an Inter-Ministerial Committee to define and develop Uganda’s UHC roadmap. The Inter-Ministerial Committee allows collaboration between multiple stakeholders from international, national, and regional partners which facilitates discussions, but also support on implementation stages with evidence-based and technical expertise. Communities’ representatives, such as the Uganda National Health Consumers’ Organisations influence the development of policy actions. To enable multi-sectoral participation, the UHC roadmap is publicly available, and it is shared with country partners.^14^ Academic experts also have a role to play. This is the case, notwithstanding the important observation of the accompanying paper, that academia and civil society have overall relatively little influence on Uganda’s policy decision-making.^1^ Academics could help scope the main barriers to achieving of UHC in Uganda, spanning human resources, infrastructure and health management information.^15^ This could help bring focus to advocacy efforts on what needs to be done to make real and sustained progress towards UHC.

## Where Next?

 Political will is often invoked to measure the quantity of desire among leaders to bring about change. (It is also sometimes invoked when governments do not want to do something). Here, as shown by Nannini and colleagues, and we have reiterated, this ‘will’ is not just an individual construct but determined by powerful and potentially modifying forces in three main areas: framing the debate, tapping political windows of opportunity and mobilising resources. Getting a solid legislative framework about these political processes is the right place to start.

## Ethical issues

 Not applicable.

## Competing interests

 Authors declare that they have no competing interests.

## Authors’ contributions

 Conception and design: DS, CE, and MB. Drafting of the manuscript: CE and MB. Critical revision of the manuscript and supervision: DS.
